# *Aspergillus awamori* Feeding Modifies Lipid Metabolism in Rats

**DOI:** 10.1155/2013/594393

**Published:** 2013-06-12

**Authors:** Ahmed A. Saleh, Akira Ohtsuka, Masahiro Yamamoto, Kunioki Hayashi

**Affiliations:** ^1^Department of Biochemical Science and Technology, Faculty of Agriculture, Kagoshima University, Kagoshima, Kagoshima 890-0065, Japan; ^2^Department of Poultry Production, Faculty of Agriculture, Kafrelsheikh University, Kafr El-Sheikh 333516, Egypt; ^3^Biogenkoji Research Institute, Mizobe kirishima 876-15, Japan

## Abstract

In the present study, an experiment was conducted to show that *A. awamori* modifies lipid metabolism in mammals. A total number of 24 rats at 6 weeks of age were divided into 2 groups (10% and 30% fat dietary groups), and each group was further divided into control and experimental groups (6 rats per group). Rats in the experimental groups were given diets containing 0.05% *A. awamori*. The diets were administered for 3 weeks to evaluate the effects of *A. awamori* on growth, plasma lipid profile, and the expressions of genes related to lipid metabolism in the liver. After the rats were fed *A. awamori*, body weight gain was increased, while food intake was decreased; therefore, food efficiency was increased in both *A. awamori* groups. Plasma triglycerides, LDL cholesterol, and glucose levels were decreased, but plasma HDL cholesterol levels were increased. Furthermore, saturated fatty acids were decreased while; unsaturated fatty acids were increased in the liver. The liver mRNA levels of FAS, ACC, delta-6-desaturase, and HMG-CoA reductase were increased, while the mRNA level of LDL receptor was decreased. From these data, it is proposed that *A. awamori* could be used as an effective probiotic to prevent lifestyle-related diseases in humans.

## 1. Introduction

It has been reported that* Aspergillus *provides beneficial effects on a host's health by affecting the host's intestinal microflora. Their beneficial effects on human health, including the alleviation of lactose intolerance, immunomodulation, hypocholesterolemic effects, and a reduction in the risk of gastrointestinal disease have been demonstrated previously [1–3]. *Aspergillus awamori* (*A. awamori*), a variant of *Aspergillus niger*, is a fungus that has long been used for food processing in Japan. The products that are processed by *A. niger* are given GRAS (Generally Recognised as Safe) status from the FDA [4]. *A. awamori* is also known to produce enzymes that enhance carbohydrates and proteins digestion [5]. Furthermore, we have reported that unsaturated fatty acid levels are increased, while saturated fatty acid levels are decreased in skeletal muscle after* A. awamori* feeding [6]. Polyunsaturated fatty acids reduce the risk of cardiovascular diseases by reducing blood lipids levels and platelet reactivity and aggregation, indicating that *A. awamori* feeding may be effective in reducing the risk of lifestyle-related diseases in humans. Here, we report that *A. awamori* modifies the plasma lipids profile and changes the liver fatty acids composition in rats with no harmful effects.

## 2. Materials and Methods

### 2.1. Animals and Diets

The animal experiment was conducted in accordance with the guidelines of Kagoshima University. 

 Twenty-four male Sprague-Dawley rats at 5 weeks of age were obtained from SLC (Shizuoka, Japan) and individually housed in stainless-steel wire-mesh cages in a temperature-controlled room at 24°C with 55% relative humidity and a 12 h light: dark cycle. The rats were allowed free access to food and water. During a 7 d prefeeding period, the rats were fed a control diet. The rats were divided into 2 groups (10% and 30% fat dietary groups), and each group was further divided into control and experimental groups (6 rats per group). Rats in the experimental groups were given diets containing 0.05% *A. awamori* (Table 1). The diets were administered for 3 weeks to evaluate the effects of *A. awamori* on growth, digestibility of energy and crude protein, abdominal fat content, organs weights, plasma lipid profile, liver fatty acids profile and the expressions of genes related to lipid metabolism in the liver. At the end of the experimental period, all rats were killed by decapitation and their organs were dissected out and used for analysis.

*A. awamori* was prepared at Bio genkoji Research Institute (Kagoshima, Japan). *A. awamori* was made as described by Yamamoto et al. [7]. Briefly, 6 tons of wheat bran and 1 ton of SDBP were mixed in a well-ventilated container with blender and were heated (100°C) and sterilized by steam. After cooling down the sterilized materials to 35°C, 6 kg of *A. awamori* spores and 70 kg of sterilized used frying oil were added. During the first 5 days of the prefermentation *A. awamori* grew fully. Thereafter, 1 ton of sterilized SDBP and 70 kg of used frying oil were added daily up to 70 days. During the fermentation, the ventilation fans were used to keep the temperature around 40°C which is the optimal temperature for *A. awamori*. In addition, the blender of the container kept working to homogenize the contents. After 70 days of the fermentation, the fermented product was cooled down by continuous ventilation. During the fermentation process, many types of enzymes are produced by the *A. awamori.* One gram of fermented product contained 2.56 U of glucoamylase, 0.05 U of a-glucosidase, 2.09 U of a-amylase, and 2345 U of acidic protease. The fungus was mixed into the diets. The numbers of *A. awamori* spores given was approximately 25 × 10^4^/g feed. 

### 2.2. Biochemical Analysis

#### 2.2.1. Blood and Liver

Blood samples were collected into heparinised test tubes, quickly centrifuged at 5,900 ×g for 10 minutes at 4°C to separate the plasma, and stored at −30°C for analysis. Total cholesterol, triglycerides, HDL, LDL, GOT, and glucose levels in the plasma were measured by an automated Fuji DRY-CHEM 3500 (Fuji Medical Systems, Tokyo, Japan) according to the manufacturer's instructions. The concentration of plasma thiobarbituric acid-reactive substance (TBARS) was measured using the method of Ohkawa et al. [8]. The plasma 3-methylhistidine concentration was measured by HPLC according to the method of Hayashi et al. [9].

#### 2.2.2. Digestibility of Energy and Crude Protein

The dietary and faecal contents of crude protein and gross energy were measured by a macrocorder machine (J-Science Lab Co., Ltd, Kyoto, Japan) and bomb calorie meter (Yoshida, Tokyo, Japan), respectively. The following calculations were made: protein digestibility = (total crude protein intake − total faecal crude protein)/total crude protein intake × 100 and energy digestibility = (total energy intake − total faecal energy)/total energy intake × 100.

#### 2.2.3. RNA Isolation and Real-Time PCR

Total RNA was extracted from a piece of liver (approximately 100 mg) using an RNeasy Fibrous Tissue Mini Kit (Qiagen, Tokyo, Japan) according to the manufacturer's protocol. The RNA concentration and purity were determined spectrophotometrically using *A*_260_ and *A*_280_ values in a photometer (BioPhotometer, Eppendorf, Hamburg, Germany). The ratio of *A*_260_/*A*_280_ for all samples was between 1.8 and 2.0. cDNA was synthesised at 400 ng RNA per 10 *μ*L of reaction solution with the PrimeScript RT reagent Kit (Perfect Real Time, Takara, Shiga, Japan) by the program temperature control system PC320 (Astec, Fukuoka, Japan), which was set at reverse transcription at 37°C for 15 min, inactivation of reverse transcriptase at 85°C for 5 s, and cooling at 4°C for 5 min. Real-time PCR primers were prepared according to the method of Nakashima [10]. Gene expression was measured by real-time PCR using the 7300 Real Time PCR system (Applied Biosystems, Foster City, CA USA) with SYBR Premix Ex Taq (Perfect Real-Time, TaKaRa). The thermal cycle was as follows: 1 cycle at 95°C for 10 s, and 60 cycles at 95°C for 5 s, 60°C for 31 s. The expression of glyceraldehyde-3-phosphate dehydrogenase (GAPDH) mRNA was used as an internal standard and was not significantly different among the experimental groups. Gene expression results are shown as % of the control value.

#### 2.2.4. Fatty Acids Analysis

Lipids were extracted from the liver with a mixture of chloroform and methanol (2 : 1) in a separatory funnel. The funnel was shaken carefully for 15 min and left to stand for 4 h to separate the organic layer. The organic layer was collected, passed through a glass funnel containing anhydrous sodium sulphate, and evaporated to near dryness using a vacuum evaporator. The extracted fat (100 mg) was placed into a 10 mL volumetric flask, and 2.5 mL of 0.5 N methanolic NaOH was added. The mixture was then heated in a steam bath for approximately 5 min. Four millilitres of BF_3_/MeOH was then added to the flask and the mixture was boiled for 2 min in a water bath. After cooling, a saturated NaCl solution was added to the mixture to reach a total volume of 8 mL. The mixture was then transferred to a separation funnel and extracted with 6 mL petroleum ether. The ether phase was then evaporated in a water bath at 60°C. The obtained methyl ester of the fatty acid fraction was dissolved in 1 mL of hexane and used for the fatty acid analysis. Fatty acids were separated by GC-MS (Thermo Fisher, MA, USA) on a capillary column (30 m × 0.25 mm i.d. DB-1 coated with a 0.25 *μ*m film of dimethyl polysiloxane) (J&W Scientific, CA, USA) using 1 *μ*L of sample. The temperature of the column was 150°C at the time of injection, then was programmed to increase 5°C min^−1^ to 250°C and was maintained at that temperature for 5 min. Injection was performed with a split ratio of 10 : 1. The flow rate was 1.0 mL min^−1^, helium was used as the carrier gas and the injector temperature was 250°C. The MS detection conditions were as follows: interface temperature, 230°C; ionisation mode, EI+; electron energy, 70 eV; full scan acquisition mode; and mass range, 33–450 amu. Fatty acids were identified using authentic standards and online NIST-library spectra. 

### 2.3. Statistical Analysis

The differences among treatment groups and control groups were analysed by the General Liner model using SPSS v. 17.0 (Statistical Packages for the Social Sciences, released August 23, 2008). The significant differences among the treatments were compared by Duncan's new multiple-range test. *P* ≤ 0.05 was set as the limit of significance.

## 3. Results

 The effects of feeding *A. awamori* on growth and digestibility in rats are summarised in Table 2. After *A. awamori* feeding, body weight gain was increased, while food intake was decreased; therefore food efficiency was increased in both *A. awamori *groups. The digestibility of protein and energy was improved by feeding *A. awamori.*

 The effect of feeding *A. awamori* on organ weights is summarised in Table 3. The weights of abdominal fat, heart, and kidney were decreased after *A. awamori* feeding, while spleen weight was not affected. Gastrocnemius muscle and liver weights were increased.

 The effect of feeding *A. awamori* on plasma GOT, TBARS, and 3-methylhistidine levels is summarised in Table 4. Plasma GOT was decreased by feeding 30% butter with 0.05% *A. awamori*. Plasma TBARS was decreased significantly by *A. awamori* feeding despite the high levels of dietary fat. Plasma 3-methylhistidine was decreased by *A. awamori *feeding.

Table 5 shows the effect of feeding *A. awamori* on the liver fatty acids profile. The levels of saturated fatty acids (palmitic acid and stearic acid) were significantly decreased while the levels of unsaturated fatty acids (oleic acid, arachidonic acid, linoleic acid, and linolenic acid) were all significantly increased by *A. awamori* feeding. 

**Figure 1:**
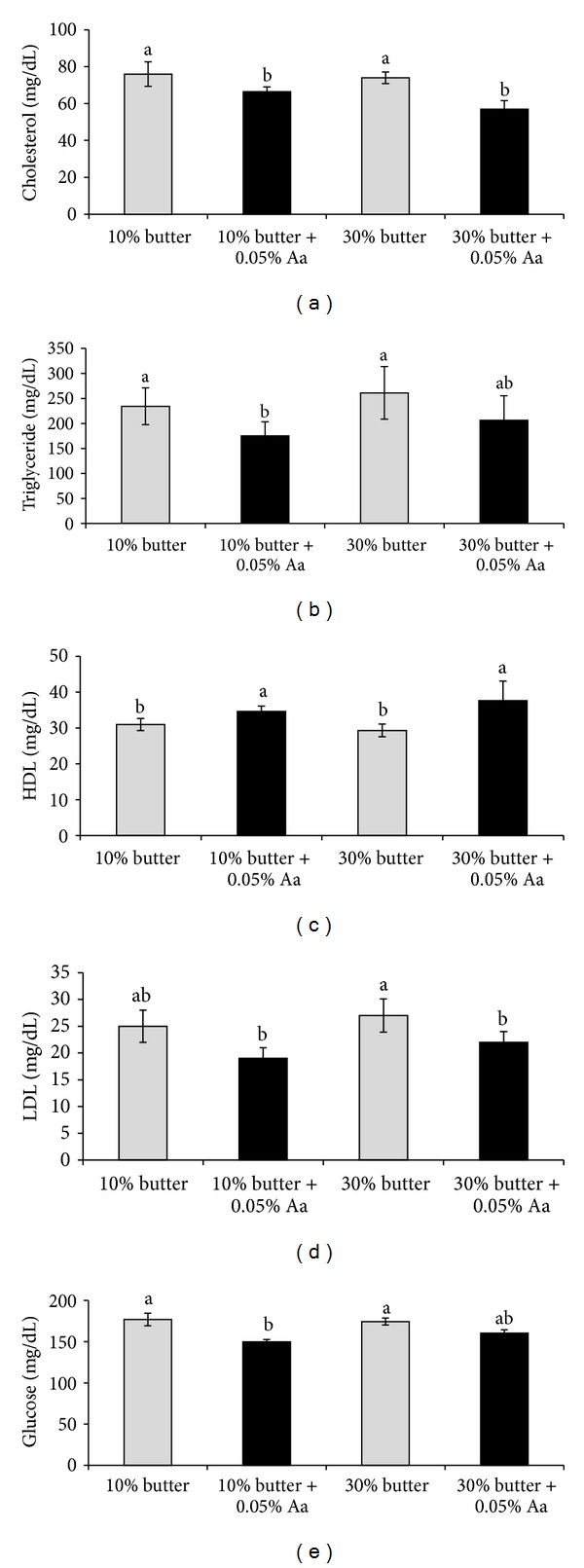
Effect of *Aspergillus awamori *feeding on the plasma levels of cholesterol (a), triglycerides (b), HDL (c), LDL (d), and glucose (e). Values are expressed as the means ± standard error. Data were analysed by two-way analysis of variance and Duncan's new multiple range tests. ^a-b^Means with different superscripts differ from each other (*P* < 0.05).

Figure 1 shows the effect of feeding *A. awamori* on plasma characteristics. Plasma cholesterol, triglyceride, LDL, and glucose levels were all decreased, while plasma HDL levels were increased by *A. awamori* feeding. 

**Figure 2:**
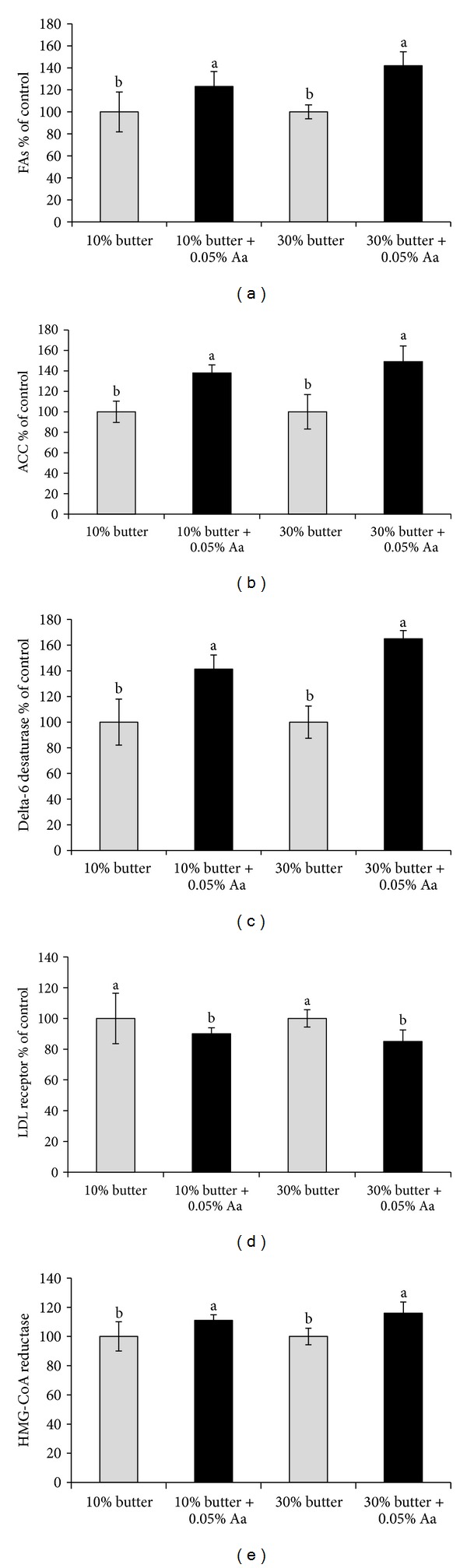
Effect of *Aspergillus awamori *feeding on the liver mRNA levels of FAS (a), ACC (b), delta-6 desaturase (c), LDL receptor (d), and HMG-CoA reductase (e). Values are expressed as % of the control values (means ± S.D); ^a-b^Means with different superscripts differ from each other (*P* < 0.05).

Figure 2 shows the effect of feeding *A. awamori* on the liver mRNA levels of fatty acid synthesis (FAS) (a), acetyl CoA carboxylase (ACC) (b), delta-6desaturase (c), LDL receptor (d), and HMG-CoA reductase (e). The mRNA levels of FAS, ACC, delta-6desaturase, and HMG-CoA reductase were all increased, while the mRNA level of the LDL receptor was decreased after *A. awamori* feeding. 

## 4. Discussion

 The major aim of the present study was to demonstrate that feeding *A. awamori* reduces the lipid levels in the abdomen and plasma and modifies the plasma lipid profile in rats with no harmful effects. Body weight gain was significantly increased (*P* < 0.05) when the rats were fed *A. awamori. *The increased weight gain and feed efficiency due to *A. awamori *may be partially due to the increase in the digestibility of energy and protein. Enzymes such as cellulase and xylanase which are required for the digestion of soluble nonstarch polysaccharides (NSPs) are produced by *A. awamori*. Enzymes contained in the fungus stimulate digestion and improve growth. Amsal found that *A. awamori *stimulates the digestion of raw starches [11]. 

 An increase in abdominal fat is associated with a higher risk of heart disease, hypertension, insulin resistance, and diabetes [12]. Iwashita et al. reported that a high-fat diet (HFD) is one of the factors that lead to obesity, and the long term-intake of HFD evokes a significant increase in abdominal fat in mammals [13]. However, our findings show that abdominal fat was decreased by *A. awamori *feeding indicating that *A. awamori* feeding might contribute to minimising the risk of heart disease in humans. 

 Muscle weight was increased after *A. awamori* feeding. This finding might be correlated with the observed changes in lipid metabolism. Yamamoto et al. reported that when diets containing 0.05% or 1% of *A. awamori* were administered, the breast muscle weight tended to increase in broiler chickens [14]. The growth-promoting effect of *A. awamori *can be explained by its effect on plasma 3-methylhistidine concentrations, as reported by Kamizono et al. and Saleh et al. [15, 16]. We used the plasma 3-methylhistidine concentration to track changes in muscle protein degradation, as reported by Nagasawa et al. [17]. The plasma 3-methylhisitidine level was decreased by *A. awamori *feeding, indicating a decreased rate of skeletal muscle protein degradation. The present results are consistent with previous results showing that *A. awamori* feeding decreases the plasma level of 3-methylhisitidine in broiler chickens [18]. The plasma 3-methylhisitidine level in this study was significantly decreased by *A. awamori* feeding, indicating a decreased rate of skeletal muscle protein degradation (Table 4). 

The concentrations of polyunsaturated fatty acids in the liver were increased by *A. awamori *feeding (Table 5). This finding is important because polyunsaturated fatty acids play important roles in reducing the incidence of lifestyle-related diseases such as coronary artery disease, hypertension, and diabetes, as well as certain inflammatory diseases such as arthritis and dermatitis, in humans [19]. Several studies have shown that oleic and linoleic acids are the most common unsaturated fatty acids that are produced by *Aspergillus* and linoleic acid is a major constituent of fungal lipids [20–23]. *Aspergillus *produces desaturase, which converts saturated fatty acids to unsaturated fatty acids [22]. Srianta reported that *Aspergillus terreus* produces linolenic acid, but the production of other polyunsaturated fatty acids has not been studied [24]. It is probable that the increases in oleic, linoleic, and linolenic acids in the muscle are a result of the intestinal activities of* A. awamori*. In the present study, liver unsaturated fatty acid levels were increased, while saturated fatty acid levels were decreased after *A. awamori* feeding. 

Plasma cholesterol, triglycerides, LDL cholesterol, and glucose levels were decreased, while plasma HDL levels were increased after *A. awamori *feeding (Figure 1). Kim et al. found that *A. oryzae *at a concentration of 0.1% in the diet significantly lowered serum cholesterol and triglyceride levels in chickens [25]. Saleh et al. [16] and Hajjaj et al. [26] found that the mechanism underlying the cholesterol-lowering effect of *Aspergillus *could be related to an inhibitor of 3-hydroxyl-3-methylglutaryl-coenzyme (HMG-CoA) reductase. It is well known that an HMG-CoA reductase inhibitor (statin) was extracted from a fungus [27]. Statin is recognised as safe and is widely used to treat patients with hypercholesterolemia [28]. An HMG-CoA reductase inhibitor that is present in *A. awamori *might be responsible for the decrease in carcass fat deposition. In addition, Aspergillus might affect fat deposition by influencing the activities of hormone-sensitive lipase and malate dehydrogenase enzyme in adipose tissues [29, 30]. 

 Acetyl CoA carboxylase and FAS are thought to play major roles in fatty acid synthesis, and delta 6-fatty acid desaturase (D6DES) plays a key role in the synthesis of polyunsaturated fatty acids (PUFAs) [31]. On the other hand, LDL receptor, which is found in the liver, is essential for producing low-density lipoprotein. In the present experiment, the levels of mRNAs related to fatty acid synthesis, including acetyl CoA carboxylase and delta 6-fatty acid desaturase, were all increased after *A. awamori *feeding (Figure 2). However, liver LDL receptor mRNA levels were decreased after *A. awamori* feeding. The present results are consistent with our previous results indicating that *A. awamori* feeding increases the mRNA levels of FAS, ACC, and delta-6 desaturase in chickens [6]. Sakuradani et al. found that *A. oryzae* produces delta-6 desaturase, which plays an important role in converting saturated fatty acids to unsaturated fatty acids [32]. 

## 5. Conclusions

We conclude that *A. awamori* modifies the plasma lipid profile and changes the liver fatty acid composition in rats with no harmful effects. Thus, *A. awamori* feeding might be effective in reducing the risk of lifestyle-related diseases in humans. 

## Figures and Tables

**Table 1:** Compositions of experimental diets.

	%			
Ingredients	10% Butter diet		30% Butter diet	
	Control	Experiment	Control	Experiment
Starch	50.00	49.95	30.00	29.95
Sucrose	10.00	10.00	10.00	10.00
Casein	20.00	20.00	20.00	20.00
Cellulose	5.00	5.00	5.00	5.00
Butter	10	10	30	30
Mineral mix^1^	3.50	3.50	3.50	3.50
Vitamin mix^2^	1.00	1.00	1.00	1.00
Choline chloride	0.2	0.2	0.2	0.2
Methionine	0.30	0.30	0.30	0.30
*Aspergillus awamori *	—	0.05	—	0.05

^1^Mineral supplied per kilogram of feed: 154 mg of Mn, 121 mg of Zn, 176 mg of Fe, 33 mg of Cu, 1.1 mg of I, and 0.7 mg of Se.

^2^Vitamin supplied per kilogram of feed: 3784 mcg of vitamin A, 0.066 mcg of vitamin D, 110.11 mcg of vitamin E, 12 mg of vitamin B_12_, 1.37 mg of retinol, 0.13 mg of cholecalciferol, 6.50 mg of riboflavin, 2.60 mg of thiamine hydrochloride, 1.30 mg of pyridoxamine hydrochloride, 0.03 mg of cyanocobalamin, 10.40 mg of D-pantothenic acid, 26.00 mg of nicotinic acid, 1.05 mg of vitamin K3, 0.52 mg of pteroylglutamic acid, 0.78 mg of choline chloride, 0.07 mg of biotin, and 2.54 g of sucrose.

**Table 2:** Effect of *Aspergillus awamori* feeding on growth and digestibility in rats.

	10% Butter		30% Butter	
	Control	Experiment	Control	Experiment
Initial body weight (g)	175 ± 3.40	175 ± 3.51	175 ± 4.11	175 ± 3.85
Body weight gain (g/21 days)	140 ± 3^c^	161 ± 3^b^	164 ± 6^b^	189 ± 5^a^
Food intake (g/21 days)	426 ± 10^a^	385 ± 12^b^	375 ± 11^b^	341 ± 8^c^
Food efficiency (%)	33 ± 1.4^c^	42 ± 1.3^b^	44 ± 1.5^a^	55 ± 1.3^a^
Protein digestibility (%)	74 ± 3^c^	80 ± 5^b^	82 ± 5^ab^	85 ± 4^a^
Energy digestibility (%)	75 ± 4^c^	83 ± 4^ab^	80 ± 3^b^	86 ± 4^a^

Values are expressed as means ± standard error. Data were analysed by two-way analysis of variance and Duncan's multiple range test. Means with different superscripts significantly differ from each other. ^a-b-c^Means with different superscripts differ from each other (*P*< 0.05).

**Table 3:** Effect of *Aspergillus awamori* feeding on organ relative weights.

	10% Butter		30% Butter	
	Control	Experiment	Control	Experiment
Abdominal fat (g/100 g BW)	3.4 ± 0.3^a^	2.1 ± 0.3^c^	3.8 ± 0.4^a^	2.8 ± 0.1^b^
Gastrocnemius muscle (g/100 g BW)	1.95 ± 0.05^c^	2.38 ± 0.09^b^	2.26 ± 0.13^b^	2.64 ± 0.06^a^
Liver (g/100 g BW)	7.7 ± 0.4^ab^	9.0 ± 0.4^a^	7.4 ± 0.4^b^	8.0 ± 0.2^ab^
Heart (g/100 g BW)	0.73 ± 0.03^a^	0.61 ± 0.01^b^	0.64 ± 0.03^b^	0.52 ± 0.02^c^
Kidney (g/100 g BW)	1.50 ± 0.07^a^	1.30 ± 0.04^b^	1.30 ± 0.07^b^	1.10 ± 0.04^b^
Spleen (g/100 g BW)	0.35 ± 0.01	0.39 ± 0.02	0.33 ± 0.01	0.34 ± 0.03

Values are expressed as means ± standard error. Data were analyzed by two-way analysis of variance and Duncan's multiple range test. Means with different superscripts significantly differ from each other. ^a-b-c^Means with different superscripts differ from each other (*P*< 0.05).

**Table 4:** Effect of *Aspergillus awamori* feeding on plasma GOT, TBARS, and 3-methylhistidine.

	10% Butter		30% Butter	
	Control	Experiment	Control	Experiment
Plasma GOT (I/U)	151 ± 5^a^	148 ± 8^a^	152 ± 6^a^	136 ± 5^b^
Plasma TBARS (nmol MDA/mL of plasma)	3.55 ± 0.1^b^	2.42 ± 0.09^c^	5.62 ± 0.14^a^	3.74 ± 0.16^b^
Plasma 3-methylhistidine (*µ*mol/mL)	17 ± 4^a^	12 ± 4^c^	14 ± 4^b^	10 ± 4^c^

Values are expressed as means ± standard error. Data were analyzed by two-way analysis of variance and Duncan's multiple range test. Means with different superscripts significantly differ from each other. ^a-b-c^Means with different superscripts differ from each other (*P*< 0.05).

**Table 5:** Effect of *Aspergillus awamori* feeding on liver fatty acid profile.

	Butter 10%		Butter 30%	
	Control	Experiment	Control	Experiment
Palmitic acid (mg/100 mg fat)	0.9 ± 0.03^c^	0.8 ± 0.03^c^	1.8 ± 0.04^a^	1.4 ± 0.02^b^
Stearic acid (mg/100 mg fat)	0.43 ± 0.01^b^	0.25 ± 0.02^c^	0.99 ± 0.04^a^	0.68 ± 0.03^b^
Oleic acid (mg/100 mg fat)	0.7 ± 0.05^c^	1.2 ± 0.11^b^	1.9 ± 0.12^ab^	2.3 ± 0.16^a^
Arachidonic acid (mg/100 mg fat)	0.89 ± 0.04^c^	1.32 ± 0.16^b^	1.44 ± 0.15^ab^	1.84 ± 0.12^a^
Linoleic acid (mg/100 mg fat)	1.6 ± 0.14^c^	2.8 ± 0.16^b^	2.9 ± 0.25^b^	4.1 ± 0.22^a^
Linolenic acid (mg/100 mg fat)	0.083 ± 0.004^d^	0.176 ± 0.006^c^	0.214 ± 0.005^b^	0.293 ± 0.002^a^

Values are expressed as means ± standard error. Data were analyzed by two-way analysis of variance and Duncan's multiple range test. Means with different superscripts significantly differ from each other. ^a-b-c^Means with different superscripts differ from each other (*P*< 0.05).
